# Treatment‐planning considerations for prostate implants with the new linear RadioCoil™ Pd103 brachytherapy source

**DOI:** 10.1120/jacmp.v6i3.2097

**Published:** 2005-08-17

**Authors:** Ali S. Meigooni, Shahid B. Awan, Venkata Rachabatthula, Rafiq A. Koona

**Affiliations:** ^1^ Department of Radiation Medicine University of Kentucky Chandler Medical Center 800 Rose Street Lexington Kentucky 40536‐0084 U.S.A.

**Keywords:** brachytherapy, linear source, Pd‐103, treatment planning, RadioCoil™

## Abstract

Recently, various linear source models, for example, Pd103 RadioCoil™, have been introduced to overcome the shortcomings of traditional “seed” type interstitial prostate brachytherapy implants, such as migration and clumping of the seeds. However, the existing prostate treatment‐planning systems have not been updated to perform dose calculation for implants with linear sources greater than 1.0 cm in length. In these investigations, two new models are developed for 3D dose calculation for a prostate implant with linear brachytherapy sources using the commercially available treatment‐planning systems. The proposed models are referred to as the linear‐segmented source (LSS) model and the point‐segmented source (PSS) model. The calculated dose distributions obtained by these models for a single linear source have been validated by their comparison with the Monte Carlo–simulated data. Moreover, these models were used to calculate the dose distributions for a multilinear source prostate implant, and the results were compared to “seed” type implants. The results of these investigations show that the LSS model better approximates the linear sources than the PSS model. Moreover, these models have shown a better approximation of the dose distribution from a linear source for 0.5 cm source segments as compared to 1.0 cm source segments. However, for the points close to the longitudinal axis of the source located outside the region bounded by the active length, both models show differences of approximately ±15%. These deficiencies are attributed to the limitations of the TG43 formalism for elongated sources.

PACS number: 87.53.‐j

## I. INTRODUCTION

The standard I125 and Pd103 “seed” type brachytherapy sources are currently being employed in interstitial permanent implants^(^ ^1^ ^,^ ^2^ ^)^ for various cancerous sites, such as the prostate. Despite the enormous success and improvements in interstitial brachytherapy, certain problems are still associated with loose seed implants, such as seed migration^(^ ^3^ ^–^ ^5^ ^)^ and seed embolization.^(^ ^6^ ^–^ ^8^ ^)^ Moreover, clumping^(^ ^9^ ^,^ ^10^ ^)^ of loose seeds during the implant results in underdosed or overdosed regions in the prostate volume.^(^ ^11^ ^)^ Visibility of existing seeds under ultrasound^(^ ^12^ ^)^ has also been an issue for many years.

In order to minimize the problems associated with conventional seed type brachytherapy sources, various pseudolinear or stranded source models, such as Rapid Strand™ (Oncura, Plymouth Meeting, PA), Readi‐Strand™, and Vari‐Strand™ (Advanced Care Medical, Inc.,

Oxford, CT) have been introduced. These pseudolinear source models are constructed by connecting a series of seeds in a linear fashion using a dissolvable tissue equivalent material. Muzio et al.^(^ ^13^ ^)^ reported that using linked seeds embedded in vicryl sutures (strands) reduces radioactive seed migration. In addition, Al‐Qaisieh et al.^(^ ^14^ ^)^ carried out a study based on 238 patients and confirmed that the use of I125 stranded seeds for prostate brachytherapy reduces evidence of seed embolization to zero.

Although stranded seeds^(^ ^8^ ^,^ ^15^ ^)^ solved the problem of seed migration to some extent, the process of seed stranding is relatively lengthy since it is performed by a second company. This process not only increases the cost of seeds but also results in delay in patient treatment. Moreover, a seven‐day stranding process of Pd103 seeds requires the production of seeds with approximately 25% higher activity to compensate for the source decay.

Encouraging clinical results^(^ ^8^ ^–^ ^13^ ^,^ ^16^ ^)^ of the stranded seeds attracted vendors to develop true linear sources. RadioMed Corporation (Tyngsboro, MA) recently introduced a linear Pd103 source called RadioCoil™. This new source is 0.35 mm in diameter and is available in integer lengths from 1.0 cm to 6.0 cm.

Despite the enormous improvements in the technical aspects of brachytherapy source design and treatment delivery, there are several shortcomings in the linear source treatment‐planning techniques. The traditional brachytherapy treatment‐planning systems^(^ ^17^ ^)^ were based on a point source approximation due to random distribution of the sources within the implant. However, this approximation is invalid for implants with linear or stranded sources. Although the implants with the flexible Ir192 wires have been around for a long time and are widely used in Europe, no simple and unified treatment‐planning technique was adapted for isodose calculations until Schlienger et al.^(^ ^18^ ^)^ presented a new method called Escargot. However, this technique is not utilized for treatment planning with low‐energy brachytherapy sources. The linear source approximation model of the AAPM Task Group 43 (TG43) protocol^(^ ^19^ ^)^ was recently implemented in the commercially available treatment‐planning systems for dose calculation with low‐energy, linear brachytherapy sources. However, there are certain limitations in their practical applications. For example, the present planning systems only assume the seed orientation^(^ ^20^ ^)^ to be perpendicular to the ultrasound or CT images. Therefore, digitizing the center of the source is the only controlled variable for the source positions. Schaart et al.^(^ ^21^ ^)^ have discussed the shortcomings of the original TG43 protocol for parameterization of long brachytherapy sources, which is an additional limitation for the treatment‐planning systems. Therefore, planning software technology requires significant improvements to be compatible with treatment technology. As an intermediate solution to the above problem, we have developed two new models of dose calculation with linear brachytherapy sources until a more permanent solution is introduced.

In this project, application of the new models for dose calculation with linear or stranded brachytherapy sources using the commercially available treatment‐planning systems was studied. These models were validated by calculating the dose distributions around RadioCoil™ linear sources using Prowess™ and VariSeed™ treatment‐planning systems, and the results were compared with Monte Carlo–simulated data.

## II. MATERIALS AND METHODS

### A. Linear source

In these investigations, the 3D dose distributions of linear sources were calculated for single and multiple RadioCoil™ source implants. These sources are fabricated from a ribbon of high purity rhodium that is activated in a cyclotron to produce radioactive palladium‐103, which is uniformly distributed throughout the ribbon. This ribbon is then turned into a coiled shape with a diameter of 0.35 mm and cut into integral lengths ranging from 1.0 cm to 6.0 cm. The apparent activity of these sources ranges from 1.0‐2.8 mCi/cm. Dosimetric characteristics of RadioCoil™ source have been investigated by Meigooni et al.^(^ ^22^ ^)^ following the updated TG43(TG43U1) recommendations.^(^ ^23^ ^)^

### B. Monte Carlo simulations

The validity of the new models was examined by comparing the dose distributions around the RadioCoil™ linear source, obtained from the treatment‐planning systems, with the published Monte Carlo–simulated data.^(^ ^22^ ^)^ In addition to the published data, a few more Monte Carlo simulations were performed using the PTRAN Monte Carlo code (v 7.43)^(^ ^24^ ^)^ with DLC 146 source library^(^ ^25^ ^)^ in liquid water and dry air media. These calculations were carried out following the same procedures and geometrical setup as described in our previous publication.^(^ ^22^ ^)^ These additional calculations were used to clarify the accuracy of superposition of the dose distribution from segmented sources as compared to that of a single linear source. Moreover, the intersource attenuation effect was also studied by simulating the active source segments in a series of nonactive source segments, as described in section D1.

### C. Treatment‐planning software

Clinical application of linear source models (longer than 1.0 cm) was tested on the Prowess™ 3.21 (Chico, CA) and VariSeed™ 7.1 treatment‐planning systems (Varian Medical Systems, Charlottesville, VA). Both of these treatment‐planning systems utilize 3D dosimetry technique to generate an overview of the dose distribution around a brachytherapy source using either line or point source approximations.^(^ ^17^ ^)^ The TG43 formalisms^(^ ^23^ ^)^ and the dosimetric characteristics of 0.5 cm and 1.0 cm long linear RadioCoil™ sources^(^ ^22^ ^)^ were used in these planning systems. It should be noted that VariSeed™ accepts all the TG43 dosimetric characteristics (dose rate constant, radial dose function, 2D anisotropy functions, 1D anisotropy function, and anisotropy constant) of a particular source in a single library. The Prowess™ treatment‐planning system incorporates these parameters into two separate libraries for point and line source approximations. However, v7.1 of the VariSeed™ treatment‐planning system does not require two different radial dose functions for point and linear source models, as recommended by the updated TG43U1. Therefore, two separate source files must be generated for these calculation methods. Both the VariSeed™ and Prowess™ treatment‐planning systems are capable of performing dose calculations using point source approximation; however, at this time, only VariSeed™ is able to use the linear source approximation. Neither of these two programs allows the overlapping of sources, independent of point or line source approximation. For example, if the dose calculation is performed with point source approximation using a 1.0 cm long source segment, the spacing between the point sources must be at least 1.0 cm. Similarly, for dose calculations with linear source approximation, the center‐to‐center spacing of the sources must be at least equal to the physical length of the source segment.

### D. Linear source models

#### D.1 Single source configuration

**Figure 1:**
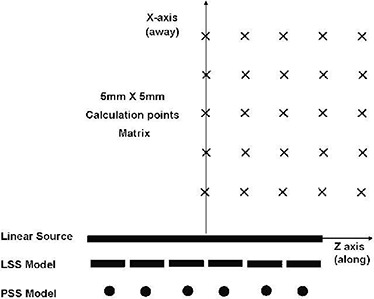
Schematic diagram of the dose calculation model used in the experimental and treatment‐planning procedures for a 3.0 cm long source. In the LSS and PSS models, a series of 0.5 cm or 1.0 cm (line or point sources) long source segments were used to represent a linear source.

In these investigations, two new models were introduced for prostate treatment planning with linear sources using available treatment‐planning systems. With these models, the existing treatment‐planning systems—such as Prowess™ and VariSeed™—could be utilized for dose calculations of prostate implants with elongated linear sources (i.e., active lengths longer than 1.0 cm). In these models, dose distribution around a linear source was calculated by superposition of dose contribution from a series of either linear‐segmented sources (LSS) or a series of point‐segmented sources (PSS) (Fig. 1). The source segments in these models consisted of either 0.5 cm or 1.0 cm long sources. For example, for the treatment planning of implants with RadioCoil™ sources, the TG43U1 dosimetric characteristics of 0.5 cm and 1.0 cm linear source segments were obtained from our previous publication.^(^ ^22^ ^)^ The results of dose calculations from these models were compared with Monte Carlo–simulated data.

The presently available treatment‐planning systems neglect the intersource attenuation effect, which is defined as the attenuation of the radiation from a one‐source segment by the other source segments. To determine the impact of the intersource effect on linear‐source dosimetry, the following calculations were performed using the LSS model with two different configurations described as below.

**Figure 2:**
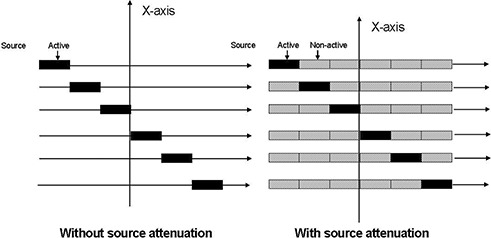
Schematic diagram of the two different six‐segmented source geometries used for verification of the effect of intersource attenuation. Solid blocks represent the active segments, and the shaded blocks represent the nonactive segments.

1. Calculations were performed using six different 0.5 cm long source segments with intersource attenuations considered (Fig. 2, right panel). The TG43U1 parameters for each of these source segments were calculated using the Monte Carlo–simulation technique (following the same procedure that we described elsewhere ^(^ ^21^ ^)^). These source configurations were designed to have an active 0.5 cm segment located between five non‐active source segments with identical source geometry. The number of nonactive segments before and after the active source segment was selected to reproduce the exact position of the active source for the LSS model in the 3 cm linear source. The TG43U1 parameters of these six different active segments were introduced into the treatment‐planning systems. The results of the linear source calculations using the LSS model with these source segments were compared to calculated dose without source attenuation.
2. Dose calculations were repeated for a 3 cm long source using six identical 0.5 cm long source segments without any intersource attenuation (Fig. 2, left panel). The parameters for these sources were obtained from our previous publications.^(^ ^22^ ^)^

#### D.2 Multisource configuration

The clinical application of the LSS and PSS models was evaluated by treatment planning of an implant with multiple linear sources. These evaluations were performed by calculating the dose distribution in a typical prostate implant patient using the VariSeed™ planning system, for a prescription dose of 125 Gy. A total of 15 RadioCoil™ Pd103 sources, comprised of two 5 cm, five 4 cm, two 3 cm, and six 2 cm sources, with 3.878 U/cm, were used in these evaluations. Each linear source was approximated by a series of 0.5 cm and 1.0 cm source segments, using the LSS and PSS models. The 3D dose distribution, dose‐volume histogram, dose to urethra, and source strength per unit length used in these calculations were compared with the results from “seed” type implant using Model $200{\;}^{103}\text{Pd}$ sources, assuming the same number of needles and prescription dose. For these comparisons, the source strengths for 0.5 cm and 1.0 cm RadioCoil™ linear source segments equivalent to that of the Model $200{\;}^{103}\text{Pd}$ source were obtained using the formalism presented by Heintz et al.^(^ ^26^ ^)^

## III. RESULTS

### A. Single‐source configuration

**Figure 3:**
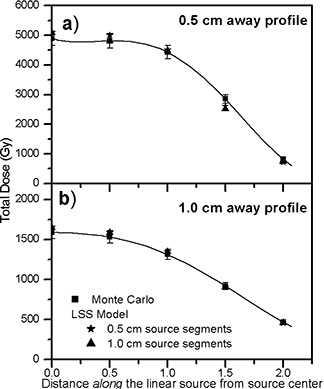
Comparisons of Monte Carlo and the LSS model (with a series of 0.5 cm, 1.0 cm long segments) calculated dose profiles at a distance of 0.5 cm (a) and 1.0 cm (b) from a 3.0 cm long source, calculated using the VariSeed™ planning system. The solid line is a fourth‐order polynomial fit to the Monte Carlo–calculated data.

(Figure 3a) shows the comparison of the Monte Carlo–calculated dose profile from a single 3.0 cm long source with the treatment‐planning values based on the LSS model, at a distance of 0.5 cm away from the source, using a series of 0.5 cm and 1.0 cm long source segments. Similarly, (Fig. 3b) shows the comparison of the dose profiles at a distance of 1.0 cm away from a 3.0 cm long source obtained from Monte Carlo–simulated data and the LSS model. The values of the dose profiles shown in these figures are the total doses calculated for total source strengths of 775.8 U (600 mCi). The magnitudes of the source strengths in these calculations were selected such that the dose values at large distances can be determined with high precision. Table 1 shows the LSS model calculated dose profiles from 0.5 cm source segments in the VariSeed™ treatment‐planning system and the percentage differences with the Monte Carlo‐simulated data (Table 3) as a function of distance along (*z*) and away (*x*) from a 3.0 cm long source. Similarly, Table 2 shows the dose profile for a 3.0 cm long source segmented by a series of 1.0 cm sources, based on the LSS model.

**Table 1:** The LSS model calculated total dose profiles of a 3.0 cm long source using 0.5 cm source segments in the VariSeed™ treatment‐planning system and their corresponding differences with the Monte Carlo–simulated values. Total source strength in each calculation method was 775.8 U.

*z* (cm)	Total dose (Gy) from LSS model with 0.5 cm source segments				Differences (%) wrt Monte Carlo calculations			
	x=0.5 cm	x=1.0 cm	x=1.5 cm	x=2.0 cm	x=0.5 cm	x=1.0 cm	x=1.5 cm	x=2.0 cm
0.0	5090	1656	698	332	4	4	4	3
0.5	5002	1590	667	317	4	4	4	4
1.0	4516	1353	567	274	2	3	4	4
1.5	2617	907	415	213	–9	–1	1	2
2.0	714	457	259	148	–14	–3	0	1

**Table 2:** The LSS model calculated total dose profiles of a 3.0 cm long source using 1.0 cm source segments in the VariSeed™ treatment‐planning system and their corresponding differences with the Monte Carlo–simulated values. Total source strength in each calculation method was 775.8 U.

*z* (cm)	Total dose (Gy) from LSS model with L.O cm source segments				Differences (%) wrt Monte Carlo calculations			
	x=0.5 cm	x=1.0 cm	x=1.5 cm	x=2.0 cm	x=0.5 cm	x=1.0 cm	x=1.5 cm	x=2.0 cm
0.0	5084	1649	694	334	4	4	3	4
0.5	4807	1588	659	314	0	4	3	3
1.0	4488	1347	562	276	1	2	3	4
1.5	2522	907	411	211	–13	–1	0	L
2.0	735	457	257	148	–11	–2	0	1

**Table 3:** The Monte Carlo–calculated total dose profiles of a 3.0 cm long source as a function of distances along (z) and away (x) from the longitudinal axis of the source. Total source strength in each calculation method was 775.8 U.

*z* (cm)	Monte Carlo–calculated total dose profile (Gy)			
	x=0.5 cm	x=1.0 cm	x=1.5 cm	x=2.0 cm
0.0	4893	1589	670	321
0.5	4812	1532	637	303
1.0	4433	1314	547	264
1.5	2852	918	411	208
2.0	817	469	257	146

**Table 4:** The PSS model calculated total dose profiles of a 3.0 cm long source based on 0.5 cm segments source data in the VariSeed™ treatment‐planning system and their corresponding differences with the Monte Carlo‐simulated values. These values are presented as a function of distances along (z) and away (x) from the longitudinal axis of the source. Total source strength in each calculation method was 775.8 U.

*z* (cm)	Total dose (Gy) from PSS model with 0.5 cm source segments				Differences (%) wrt Monte Carlo calculations			
	x=0.5 cm	x=1.0 cm	x=1.5 cm	x=2.0 cm	x=0.5 cm	x=1.0 cm	x=1.5 cm	x=2.0 cm
0.0	5032	1545	636	298	3	–3	–5	–8
0.5	4916	1483	608	286	2	–3	–5	–6
1.0	4390	1269	523	249	–1	–4	–5	–6
1.5	2661	882	393	198	–7	–4	–5	–5
2.0	922	488	259	142	11	4	0	–3

(Figures 4a) and (b) show the comparison of the dose calculations of a 3.0 cm long source using the PSS model, in the VariSeed™ planning system, with the Monte Carlo–simulated data at distances (i.e., away from the source) of 0.5 cm and 1.0 cm, respectively. Tables 5 and 6 show the dose profiles of a 3.0 cm long source calculated with the PSS model, using 0.5 cm and 1.0 cm source segments and their percentage differences with the Monte Carlo‐simulated data (Table 3). Similar results were obtained using the PSS model in the PROWESS™ (v3.21) treatment‐planning system (Fig. 5, Tables 7 and 8). The PSS calculated values for other source lengths have indicated similar results.

**Figure 4:**
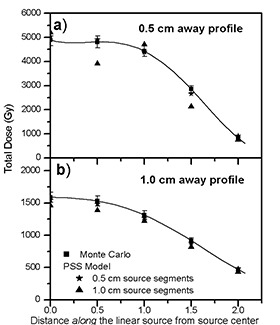
Comparisons of Monte Carlo and the PSS model (with a series of 0.5 cm, 1.0 cm long segments) calculated dose profiles at a distance of 0.5 cm (a) and 1.0 cm (b) from a 3.0 cm long source, calculated using the VariSeed™ planning system. The solid line is a fourth‐order polynomial fit to the Monte Carlo–calculated data.

**Figure 5:**
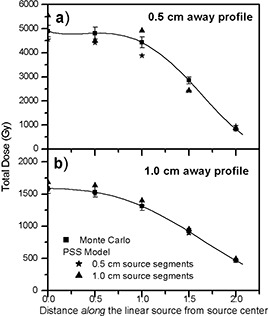
Comparisons of Monte Carlo and the PSS model (with a series of 0.5_cm, 1.0 cm long segments) calculated dose profiles at a distance of 0.5 cm (a) and 1.0 cm (b) from a 3.0 cm long source, calculated using the_Prowess™ planning system. The solid line is a fourth‐order polynomial fit to the Monte Carlo–calculated data.

**Table 5:** The PSS model calculated total dose profiles of a 3.0 cm long source based on 1.0 cm segments source data in the VariSeed™ treatment‐planning system and their corresponding differences with the Monte Carlo‐simulated values. These values are presented as a function of distances along (z) and away (x) from the longitudinal axis of the source. Total source strength in each calculation method was 775.8 U.

z (cm)	Total dose (Gy) from PSS model with 1.0 cm source segments				Differences (%) wrt Monte Carlo calculations			
	x=0.5 cm	x=1.0 cm	x=1.5 cm	x=2.0 cm	x=0.5 cm	x=1.0 cm	x=1.5 cm	x=2.0 cm
0.0	5205	1464	604	286	6	–9	–11	–12
0.5	3913	1389	577	273	–23	–10	–10	–11
1.0	4701	1220	496	239	6	–8	–10	–11
1.5	2129	820	370	187	–34	–12	–11	–11
2.0	748	434	239	133	–9	–8	–8	–10

**Table 6:** The PSS model calculated total dose profiles of a 3.0 cm long source using 0.5 cm segments source data in the PROWESS™ treatment‐planning system and their corresponding differences with the Monte Carlo‐simulated values. These values are presented as a function of distances along (z) and away (x) from the longitudinal axis of the source. Total source strength in each calculation method was 775.8 U.

*z* (cm)	Total dose (Gy) from PSS model with PROWESS™				Differences (%) wrt Monte Carlo calculations			
	x=0.5 cm	x=1.0 cm	x=1.5 cm	x=2.0 cm	x=0.5 cm	x=1.0 cm	x=1.5 cm	x=2.0 cm
0.0	4547	1569	627	300	–8	–1	–7	–7
0.5	4412	1508	606	288	–9	–2	–5	–5
1.0	3880	1316	525	250	–14	0	–4	–6
1.5	2420	894	390	197	–18	–3	–6	–6
2.0	951	464	249	141	14	–1	–3	–3

**Table 7:** The PSS model calculated total dose profiles of a 3.0 cm long source based on 1.0 cm segments source data in the PROWESS™ treatment‐planning system and their corresponding differences with the Monte Carlo–simulated values. These values are presented as a function of distances along (z) and away (x) from the longitudinal axis of the source. Total source strength in each calculation method was 775.8 U.

*z* (cm)	Total dose (Gy) from PSS model with PROWESS™				Differences (%) wrt Monte Carlo calculations			
	x=0.5 cm	x=1.0 cm	x=1.5 cm	x=2.0 cm	x=0.5 cm	x=1.0 cm	x=1.5 cm	x=2.0 cm
0.0	5545	1683	664	313	12	6	–1	–3
0.5	4498	1633	619	276	–7	6	–3	–10
1.0	4925	1400	546	259	10	6	0	–2
1.5	2436	952	399	192	–17	3	–3	–8
2.0	867	494	257	149	6	5	0	2

The impact of intersource attenuation on the LSS Model calculated dose profiles at 0.5 cm and 1.0 cm distances from a 3 cm long source is presented in Table 4. This Table indicates the percentage differences between the Monte Carlo simulated dose profile from a 3 cm linear source and the LSS‐Model calculated data, with and without intersource attenuation. The LSS‐Model calculations were performed using 0.5 cm source segments.

### B. Multisource configuration

**Figure 6:**
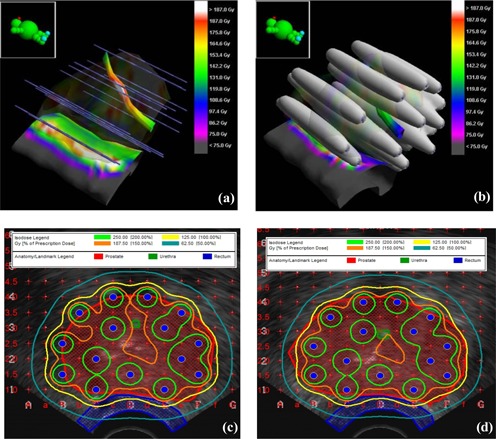
Clinical application of the_LSS model with existing treatment‐planning systems using a 0.5 cm RadioCoil™ linear source segments for a sample prostate cancer patient. Three –dimensional views of the patterns of needles and dose distribution at the vicinity of the sources ((a)and (b)). Dose_distributions of the prostate implant calculated in two different axial views_((c) and (d)).

The clinical applications of the LSS and PSS models were examined by dose calculation in a sample prostate cancer patient. (Figures 6a) and (b) show the 3D views of the patterns of needles and dose distribution at the vicinity of the sources, respectively, indicating uniform dose distribution along each needle. (Figure 6b) also indicates that the dose calculations based on 0.5 cm source segments is a good representation of a linear source in each needle. (Figures 6c) and (d) show the dose distributions in two different axial images of the prostate gland. These figures indicate a good coverage of the prostate gland. The quantitative comparisons of the volumes of different isodose lines calculated with the LSS and PSS models for the 0.5 cm and 1.0 cm RadioCoil™ source segments with model 200, Pd103 source are shown in Tables 9 and 10. In addition, these tables show the comparisons between dose values covering the 90%, 50%, and 10% of the prostate gland, using both the RadioCoil™ and Model $200{\;}^{103}\text{Pd}$ sources. Total source strengths of 186.14 U, 191.80U, and 204.76U were used for calculations with Model 200, 0.5 cm and 1.0 cm RadioCoil™ source segments, respectively, for a prescription dose of 125 Gy.

**Table 8:** The quantitative values of the volumes and dose coverage calculated using the LSS model for the RadioCoil™ wires and the Model 200, Pd103 sources with linear source approximation.

	LSS model (line source approximation)				
	Model 200	0.5 cm Linear source		1.0 cm Linear source	
	Volume (cm^3^)	Volume (cm^3^)	% Difference to Model 200	Volume (cm^3^)	% Difference to Model 200
V200	12.70	11.29	12	11.58	10
V150	26.05	24.49	6	24.68	6
V100	32.23	32.07	0	32.11	0
V50	32.57	32.57	0	32.57	0
	Dose (Gy)	Dose (Gy)	% Difference to Model 200	Dose(Gy)	% Difference to Model 200
D90	168.18	162.06	4	162.78	3
D50	231.86	222.78	4	223.82	4
D10	470.50	434.88	8	448.63	5

**Table 9:** The quantitative values of the volumes and dose coverage calculated using the PSS model for the RadioCoil™ wires and the Model 200, Pd103 sources with point source approximation.

	PSS model (point source approximation)				
	Model 200	0.5 cm Linear source		1.0 cm Linear source	
	Volume (cm^3^)	Volume (cm^3^)	% Difference to Model 200	Volume (cm^3^)	% Difference to Model 200
V200	12.98	11.89	9	11.20	16
V150	25.87	24.56	5	23.85	8
VI 00	32.22	32.07	0	32.04	1
V50	32.57	32.57	0	32.57	0
	Dose (Gy)	Dose (Gy)	% Difference to Model 200	Dose(Gy)	% Difference to Model 200
D90	167.73	162.11	3	159.87	5
D50	232.50	224.57	4	220.10	6
DIO	469.99	442.19	6	446.26	5

## IV. DISCUSSION AND CONCLUSION

Two new dose calculation models have been introduced here to overcome the limitations of the current prostate brachytherapy treatment‐planning systems for dose calculations around linear sources longer than 1.0 cm. These models allow calculation of dose distribution around a linear source using either a series of linear‐segmented sources (LSS) or a series of point‐segmented sources (PSS). Dosimetric characteristics of the 0.5 cm and 1.0 cm source segments were obtained from our previously published data,^(^ ^22^ ^)^ which were determined according to the updated TG43U1 recommendations.^(^ ^23^ ^)^ These new models were implemented in dose calculations around various source lengths using two different commercially available treatment‐planning systems. The results of these calculations were compared with the Monte Carlo–simulated data in order to validate the accuracy of the new models.

The results shown in Tables 1, 2, 3 and Fig. 3 indicate that, for the points within the active boundary of a 3.0 cm long source, the LSS model, with 0.5 cm and 1.0 cm source segments, closely (within 4%) reproduces the Monte Carlo–simulated data of a given linear source. In addition, for the points outside of the region bounded by the active length of the source, when x≥1 cm (i.e., x≥1/2 of active length), the agreements between the LSS model and the Monte Carlo simulation were within 4%. However, the differences increased up to 14% for the points very close to the longitudinal axis of the source and with x<1 cm (i.e., x<1/2 of active length). These differences could be attributed to the following: (1) accuracy of the algorithm and methodology of interpolations in treatment‐planning systems and (2) limitations of the TG43U1 formalism and parameters for the elongated sources. The differences were further increased (up to 21%) when the source attenuations (Fig. 2) were incorporated in the calculations (Table 4).

The accuracy of the interpolation algorithm in the planning systems was verified by calculating the dose profile using an independent technique. This verification was performed by introducing the TG43 algorithms and source parameters for the 0.5 cm source segments^(^ ^22^ ^)^ in a Microsoft Excel spreadsheet. The radial dose function and 2D anisotropy functions were introduced in the form of polynomial coefficients, for ease of interpolation technique. Dose values were calculated by superposition of the dose contributions from each source segment for the same points as those in the treatment‐planning systems. The results of these calculations were compared to the Monte Carlo–simulated values as well as the data obtained from the treatmentplanning systems. These comparisons showed no significant differences (less than 1%) between this independent method and treatment‐planning data. Therefore, the interpolation techniques of the treatment‐planning systems were not contributing to the discrepancies between the Monte Carlo–simulated data and treatment‐planning values. Moreover, it was found that the discrepancies always increase for the points at small angles immediately after the active length of the source, where the variation of the anisotropy function is the maximum. This discrepancy could be attributed to the deficiency of the TG43 formalism for defining the anisotropy and geometric function of the elongated source.

The results of these investigations show that the PSS model underpredicts the Monte Carlo–calculated values up to 8% for 0.5 cm source segments and 12% for 1.0 cm source segments, for the points with x≥1.0  cm. The disagreements increased for the points closer to the longitudinal axis of the source, particularly when larger source segments were used. These differences would further increase if the intersource attenuation is incorporated in the PSS model. The larger discrepancies of the PSS model compared to the LSS model could be attributed to the fact that the concept of point source approximation was based on random orientation of the seeds within the implant volume. Therefore, for a fixed source orientation, this model leads to underdose or overdose regions, which are shown in Tables 5 to 8.

The results of multisource calculations show the clinical application of the new models with existing prostate treatment‐planning systems. The qualitative and quantitative evaluation of these results indicates the practical application of this intermediate solution for dose calculation for prostate implants with linear brachytherapy sources. Tables 9 and 10 show the quantitative comparison between the volume of various isodose lines as well as the dose coverage for a typical prostate implant. These results indicate that values of the volume of the 100% dose (V100) calculated with both the LSS and the PSS models using the RadioCoil™ wires are in excellent agreement (within 1%) with the Model $200{\;}^{103}\text{Pd}$ source. However, the LSS model shows differences of up to 6% for V150% and 12% for V200, for both 0.5 cm and 1.0 cm source segments, as compared to the Model 200, Pd103 source. The V150 values, calculated with the PSS model, using 0.5 cm and 1.0 cm source segment, were found to be 5% and 8%, different from that of Model 200, Pd103 source. Moreover, the doses to the 90% (D90) and 50% (D50) of the prostate gland, calculated with RadioCoil™ wires were within 4% for the LSS model and up to 6% for the PSS model, as compared to those of the Model $200{\;}^{103}\text{Pd}$ source.

In summary, the results of these investigations show that both the LSS and PSS models can easily be adapted for the present treatment‐planning system for prostate implant dose calculations. These results indicate that for the 0.5 cm source segment, the LSS and PSS models are in good agreement (less than 5% and 8%, respectively) with that of Monte Carlo–simulated data. However, the LSS model gives better agreement than the PSS model. Use of 1.0 cm source segments in the PSS model leads to larger discrepancies, whereas the LSS model closely represents the dose profile of a linear source. Therefore, the use of 1.0 cm source segment in the PSS model is not recommended. The limitation of the LSS model is mainly for the points outside the active length and very close to the longitudinal axis of the source. This limitation is attributed to the deficiency of the anisotropy function defined by the TG43 protocol, as applied for the elongated source. The required modifications to the TG43 formalism for the elongated source are under investigation at our institution, and the results will be presented in forthcoming publications.

## ACKNOWLEDGMENT

This work was partially supported by the U.S Army Medical Research under DAMD 17‐02‐1‐0242. The authors would like to express their gratitude to Dr. Piran Sioshansi and Mr. Ray Bricault for providing us the opportunity to work on this project.
